# 110. MRSA nasal swab as a stewardship tool to guide IV Vancomycin in diabetic foot infections

**DOI:** 10.1093/ofid/ofab466.312

**Published:** 2021-12-04

**Authors:** Alex Lazo-Vasquez, Michael Piazza, Leopoldo Cordova, Lauren Bjork, Rolando A Zamora Gonzalez, Paola Lichtenberger, Paola Lichtenberger, Gio Baracco

**Affiliations:** 1 University of Miami Hospital / Jackson Memorial, Miami, Florida; 2 University of miami / jackson memorial Hospital, Miami, FL; 3 Miami Veterans Affairs Healthcare System, Miami, Florida; 4 University of Miami / Jackson Memorial Hospital, Miami, Florida; 5 University of Miami MIller School of Medicine, Miami, FL; 6 University of Miami Hospital / Jackson Hospital / Miami VA Hospial, Miami, Florida

## Abstract

**Background:**

The Infectious Disease Society of America (IDSA) guidelines suggest empiric Methicillin-Resistant Staphylococcus Aureus (MRSA) coverage for Diabetic Foot Infection (DFI) with a history of MRSA infection, if local prevalence is high, or if the infection is severe. However, data suggests that there is overutilization of vancomycin in this population and this medication is associated with toxicity. MRSA nasal screen has a high negative predictive value (NPV) for ruling out MRSA in pneumonia and other sites. We performed a medication utilization evaluation (MUE) for Vancomycin IV in DFI patients who had an MRSA nares screen to determine our own NPV of this test and feasibility to use it as an antibiotic stewardship program (ASP) tool to guide vancomycin use in this population.

**Methods:**

We retrospectively reviewed 224 patients from January 2015 to January 2020 who had a diagnosis of DFI and an MRSA nasal screen. 139 patients had cultures done. For the NPV, we excluded patients who had any MRSA positive culture or screen up to a year from admission (Figure 1).

Figure 1. Flowchart from our medication utilization evaluation showing patient’s distribution by MRSA-screen result

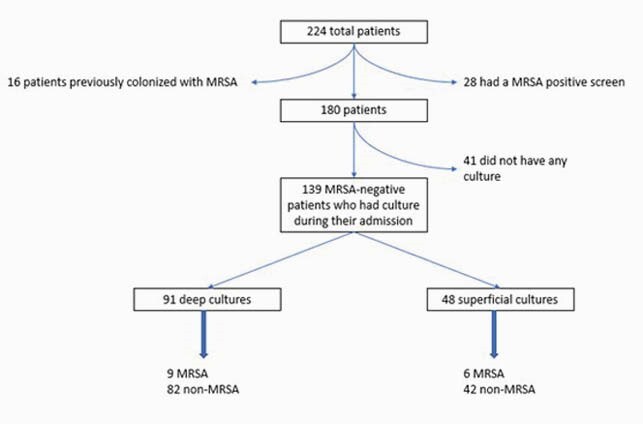

**Results:**

We found 148 (66%) patients with DFI who had received IV vancomycin empirically during the admission and 196 of them were MRSA-nares negative (Figure 2). The average days of therapy (DOT) in the MRSA-nares negative patients was 5.2 days vs 4.8 in the MRSA-nares positive patients. Out of the 139 patients with a negative MRSA nasal swab, 124 had no MRSA in cultures, yielding an NPV of 89%. If we considered only the deep cultures, the NPV increased to 90%.

Figure 2. Number of patients who received IV vancomycin grouped by MRSA-screen result

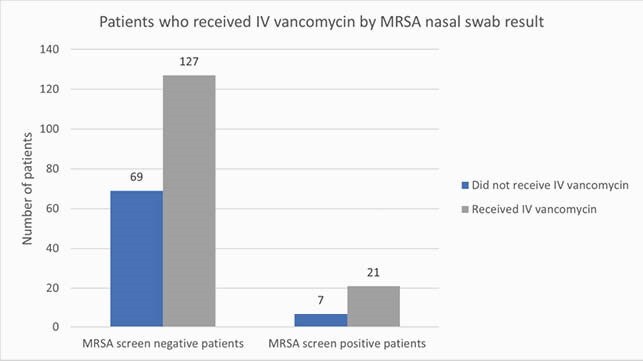

**Conclusion:**

We identified overutilization of IV vancomycin in patients with a diagnosis of DFI in our institution. Also, our NPV of the MRSA-nasal screening to rule out MRSA infection in DFI was high at 89% similar to previous studies. Based on these findings, we plan to implement a local ASP protocol (Figure 3) using MRSA nasal swab screen to decrease the empiric use of vancomycin. The results of these efforts will be analyzed and published in future iterations with the hopes to share this knowledge to reduce the use of IV vancomycin in this population in other centers.

Figure 3. Protocol draft to be used as an ASP tool to guide IV vancomycin de-escalation based on MRSA-nasal screen for DFI patients

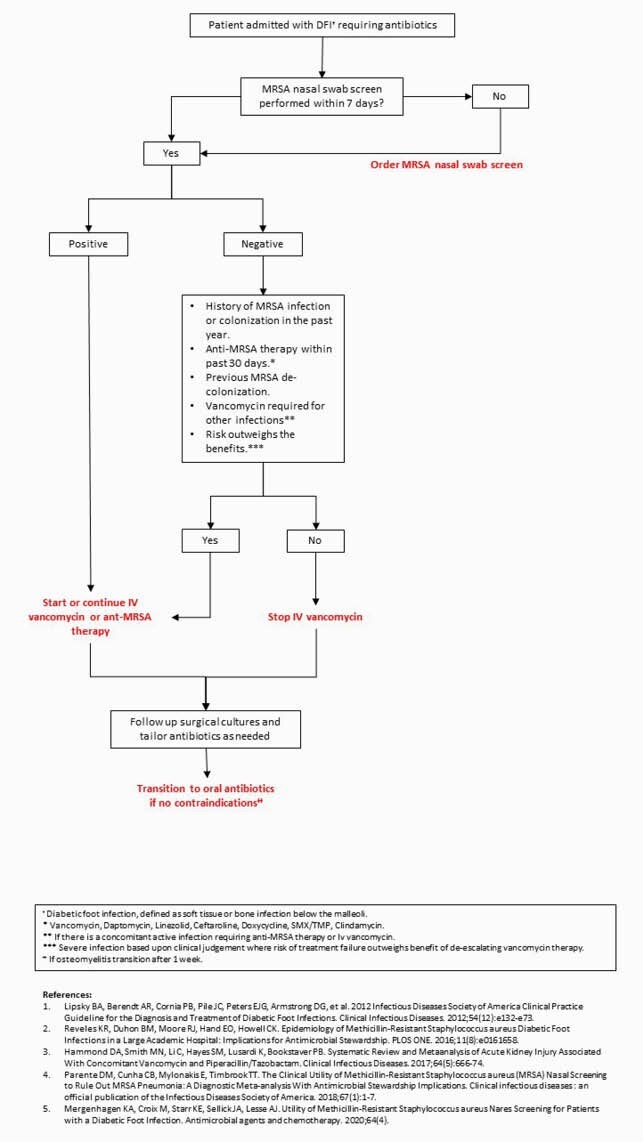

**Disclosures:**

**All Authors**: No reported disclosures

